# Neopentyl Glycol as Active Supporting Media in Shape-Stabilized PCMs

**DOI:** 10.3390/ma12193169

**Published:** 2019-09-27

**Authors:** Angel Serrano, Jean-Luc Dauvergne, Stefania Doppiu, Elena Palomo Del Barrio

**Affiliations:** 1 CIC energiGUNE, Albert Einstein 48, Miñano, 01510 Álava, Spain; aserrano@cicenergigune.com (A.S.); sdoppiu@cicenergigune.com (S.D.); 2 Ikerbasque Foundation, CIC energiGUNE, Albert Einstein 48, Miñano, 01510 Álava, Spain; epalomo@cicenergigune.com

**Keywords:** shape-stabilized phase change material, Neopentyl Glycol, Docosane, expanded graphite, thermal energy storage, solid-solid PCM

## Abstract

The present work explores the feasibility of using polyalcohols with solid-solid phase transition as active supporting matrix of n-alkanes in shape-stabilized phase change materials (SSPCMs). It is well-established that the use of SSPCM avoids leakage and increases stability and easy handling of solid-liquid PCMs. Nevertheless, the resulting composite exhibits a loss of heat storage capacity due to the volume occupied by the supporting material, which does not contribute to latent heat storage. Therefore, the objective of this work is to combine solid-liquid PCMs (alkanes) with solid-solid PCMs (polyalcohols), both exhibiting a phase transition in the same range of temperature, to obtain high energy density SSPCMs. Towards that goal, the performance of Neopentyl Glycol (NPG) and Docosane as a new energetic SSPCM has been proved. The NPG-Docosane chemical compatibility and its outstanding wettability facilitate the propitious association of both materials. The higher capillary forces obtained by decreasing the NPG crystal size together with the addition of expanded graphite (EG) allowed to obtain a maximum Docosane content of 60 wt%. The addition of EG improves the shape stability at the time that increases the heat transfer properties of the composites. The analysis showed that the components of the obtained SSPCMs are able to combine their latent heats, achieving a maximum value of 210.74 J/g for the highest Docosane content. This value is much higher than those latent heats exhibited by existing SSPCMs in the same working temperature range.

## 1. Introduction

Thermal energy storage (TES) is doomed to have a key role in the energy transition in our society, both in the energy generation and in industrial, building, and automobile sectors. Among existing TES systems, the latent heat TES technologies based on Phase Change Materials (PCMs) are particularly attractive for applications where thermal energy has to be stored/delivered over a narrow temperature range or when compactness is a premium. Indeed, PCMs are able to absorb or release great amounts of energy in the form of latent heat during phase transitions at nearly constant temperature [1].

Based on their aggregation state change, three different PCMs are sorted: liquid-gas, liquid-solid, and solid-solid PCMs. Despite their high latent heat, liquid-gas PCMs are inoperative for practical purposes because of the large volume required in the storage system device [2].

Due to their high energy storage capacity and limited volume variation during phase change, solid-liquid PCMs are the most widely used materials in latent heat storage technologies. Paraffin, fatty acids, glycols, or inorganic salts are among the main compounds indexed in this group [3]. Nevertheless, the leakage of the liquid phase of these materials at temperatures above their melting point hampers their application. In that way, prior to their implementation in the final appliance, solid-liquid PCMs are usually confined in a supporting matrix or encapsulated, either macro or microstructures, which implies considerable production costs [4].

There are various techniques to embed the solid-liquid PCM into a carrier material. Microencapsulation is one of the most widespread method used to stabilize the PCM due to its high versatility through the selection of different shell materials. The current microencapsulation techniques for producing particles between 100 to 5000 μm allow to obtain PCM contents in the range of 40–75 wt% [4,5].

The use of additional stabilizer materials is another relevant option to avoid the leakage of the PCMs, giving rise to the composites known as shape-stabilized PCMs (SSPCMs). These compounds have the ability to keep the shape of the PCMs even when its temperature is above the melting point (liquid state) [6,7]. SSPCMs can be classified into two major groups, depending on the nature of the supporting material: (i) SSPCMs with supporting organic material based on polymers, which are obtained by a blending process and characterized by a continuous polymeric structure that encloses the PCM [8,9]; and (ii) SSPCMs with supporting inorganic porous materials, that are produced by incorporating liquid PCM into the porous structure by impregnation or vacuum assisted infiltration method, among others [7].

The maximum amount of embedded PCM obtained with these methods reaches values around 70 wt% by using polymer matrices [10] and up to 80–85 wt% or even higher with inorganic supporting materials [11,12,13]. However, in spite of the high PCM contents, the nanoconfinement effects of the porous support on the thermal properties of the confined PCMs usually reduce the TES capacity of the embedded PCM [14].

Taking into account these maximum PCM contents and the use of inactive carrier materials for storing energy, both microencapsulation and shape-stabilization lead to loss of heat storage capacity (often larger than 20%), due to the volume occupied by the supporting material which is needed to guarantee the stability of the composite and avoid leakage, but which does not contribute to latent heat storage. The above problems could be solved by using solid-solid PCMs. Nevertheless, their commonly lower heat of phase transitions compared with solid-liquid PCMs [15] hamper their use on their own.

The aforementioned drawback can be addressed by using an active carrier element in the SSPCMs that stores latent heat in the same PCM working temperature range than the presented by the solid-liquid PCM. In this vein, Chen et al. prepared different form-stable composites using polyurethane PCM (PUPCM) as supporting matrix with solid-solid phase transition, containing up to 25 wt% of paraffin (n-octadecane and n-eicosane) [16]. They synthetized PUPCMs via bulk polymerization containing soft segments of PEG (4000, 6000 and 10,000 g/mol of molecular weight) and 25 wt% of paraffin entrapped into the PUPCM framework, achieving 141.2 J/g and transition temperatures between 20 to 65 °C when n-eicosane was incorporated.

Going beyond this concept, the present work explores the viability to use polyalcohol with solid-solid phase transition as the active supporting matrix combined with n-alkanes, specially taking into account the great advantages that these materials exhibit. Polyalcohols often undergo solid-solid phase transitions from a layered or chained low temperature structures to a high temperature homogeneous face centered cubic crystal. The solid-solid phase transition in these compounds takes place at temperatures between 40 °C and 188 °C and it is characterized by an unusually large enthalpy of transition (110 J/g to 300 J/g) due to the effect of hydrogen-bonds [15]. Moreover, binary and ternary mixtures of these compounds often allow to obtain new solid-solid PCMs with “tailor made” energy storage properties [17]. The transition temperature, as well as the corresponding latent heat, are hence easily adjustable between those of the pure constituents. Furthermore, polyalcohols are renewable, non-toxic, non-flammable and compatible with metals and plastics [18].

On the other hand, alkanes covering a range from C_20_ to C_100_ display melting temperature between 40 °C to 115 °C with latent heats ranging from 200 J/g to 300 J/g. Besides, many binary alkane systems show solid-state solutions with very narrow temperature window (2–5 °C). As already mentioned, this property allows a blend to be tailored to a particular melting range [19].

Within the same range of phase transition temperatures, alkanes usually have higher latent heat and lower cost than polyalcohols. The objective of combining them is, therefore, to increase the energy density of polyalcohols while lowering the cost and preserving the advantages of SSPCMs.

In order to demonstrate the viability of this concept, different Neopentyl Glycol (NPG)-Docosane composites with alkane content from 40 to 70 wt% were manufactured. NPG and Docosane were selected due to their similar range of phase transition temperatures (30 to 50 °C). The NPG crystal size was controlled by two milling methods, mortar and ball-milling process and its effect on the stabilization of docosane was studied. Moreover, the addition of particles of expanded graphite (EG) as binder and heat transfer booster was also accomplished, studying its effect on the properties of the final composite. The shape-stability of the obtained composites was tested, selecting the ones without paraffin leakage. In the same way, their thermal stability was examined by thermogravimetric analysis. XRD analysis was employed in order to ensure the chemical compatibility of the raw materials. The inner structure of the selected SSPCMs was analyzed, as well as their densities and thermal properties, focusing on thermal conductivities and thermal diffusivities, as well as their thermodynamic properties such as transition temperatures and latent heats.

## 2. Materials and Methods

### 2.1. Materials

Neopentyl Glycol (NPG) and Docosane at high purity grades (99%) were supplied by Sigma-Aldrich and used as received, the main properties of both materials are shown in Table 1, where *T_t_* is the transition temperature, ∆*H* its related enthalpy change and *ρ* and *k* the density and thermal conductivity, respectively.

NPG is one of the molecules known as plastic crystal (2,2-dimethyl-1,3-propanediol) with a solid-solid phase change from an ordered low-temperature monoclinic phase (phase II) to an orientationally disordered Face Centered Cubic (FCC) phase (phase I) [22]. Highly conductive expanded graphite powder has been supplied by the company SGL Carbon SE. SIGRATHERM^®^ GFG75 was the selected *EG*, with an average particle size D_50_ of 75 μm and a powder density of 0.120 g/cm^3^.

### 2.2. SSPCM Preparation

Two kind of NPG samples were obtained as function of the ground process used for controlling its crystal size. Mortar and ball-milling processes were the methods selected for crystal size reduction, in pursuit of a higher uptake of Docosane by NPG. The use of mortar (and pestle) consists in a simple hand-grinding process, whereas in ball-milling 4 g of NPG were ground by using 4 metallic balls of 1 g during 15 min in a 8000M Mixer/Mill^®^ High-Energy Ball Mill, from SPEX SamplePrep LLC. NPG obtained by both processes are hereinafter referred to as *NPG_HG_* and *NPG_BM_*, respectively.

For the preparation of the SSPCMs, solid powder of the selected type of NPG crystals (*NPG_HG_* or *NPG_BM_*) and the proper amount of Docosane (Docosane content was varied from 40 to 70 wt% with respect to the final composite mass) were mixed and heated up to 60 °C in a hot plate with a constant stirring rate of 150 rpm during 30 min. After that time, the mixture at 65 °C was discharged in a plastic mold with 9 mm of diameter and slightly pressed, avoiding the presence of air bubbles. The composites were cooled down at room temperature for 24 h before its removal from the mold, resulting in an NPG structure enclosing Docosane. The final pellets were obtained by cutting the cylindrical composite each around 5 mm of length.

In order to study its effect on the stabilization and thermal properties of the composites, a series of SSPCMs containing expanded graphite particles were manufactured following the same mentioned method. *EG* powder was mixed together with *NPG_BM_* and Docosane previous to the heating step. In all the cases the amount of added EG in the final composite was 2.5 wt%.

Therefore, samples are hereinafter denoted according to their composition as *HG* for composites using *NPG_HG_*, *BM* for samples having *NPG_BM_* and *EG* for the composites containing expanded graphite, following by their Docosane content as percentage in weight with respect to the total composite mass. As an example, *EG_50_* is composed by a 50 wt% of Docosane, 2.5 wt% of *EG* and 47.5 wt% of *NPG_BM_*.

### 2.3. Composite Characterization

#### 2.3.1. NPG Crystal Size

The NPG crystal size distribution achieved with each milling method was studied with an optical microscope ZEISS AXIO SCOPE.A1. From these pictures the NPG crystal size distribution was calculated for each case by using the software ImageJ2.0 [23]. At least 100 crystal length measurements were collected for each sample.

#### 2.3.2. NPG-Docosane Wettability

The affinity between NPG and Docosane was studied through the contact angle of a liquid docosane drop on a NPG solid surface. A cylindrical pellet of NPG (5 mm height, 13 mm diameter) was obtained by pressing powder of NPG in a hydraulic press at 5 t in order to ensure a flat surface. The NPG pellet was placed on a heating plate and heated up to 70 °C for avoiding the solidification of the liquid docosane drop on its surface. Once the proper temperature was reached a drop of melted docosane was located on the NPG surface, taking a picture of the system at this moment. The contact angle was analysed with the software ImageJ2.0 and the plugin “*Contact angle*” [24].

#### 2.3.3. Leakage Test

To verify the ability of SSPCMs to avoid the leakage of liquid Docosane 3 cylindrical samples (5 mm height, 9 mm diameter) of each composite were placed on a heating plate and heated up to 70 °C (30 °C higher than the melting point of Docosane), keeping the final temperature constant during 1 h. During the heating process, the samples were photographed to check the shape-stability. Moreover, the samples were weighed before and after the test in order to obtain the percentage of Docosane released from the composites.

#### 2.3.4. X-ray Diffraction (XRD)

X-ray powder diffraction measurements were carried out to investigate the solid phases of NPG, Docosane and SSPCMs. Diffraction patterns were obtained with a Bruker D8 Advance diffractometer equipped with an LYNXEYE detector using CuKα radiation (wavelength 1.5419 Å). Patterns were recorded at a tube voltage of 40 kV and a tube current of 40 mA applying a step size of 0.02° 2θ in the angular range of 10 to 80° 2θ at 1.03 s per step. Measurements were performed at room temperature.

#### 2.3.5. Scanning Electron Microscopy (SEM)

The samples were imaged by means of a scanning electron microscope Quanta 200 FEG operated in low vacuum mode at 10 kV featured with a backscattered electron detector (BSED) and large field low vacuum detector (LFD). The operational conditions were carefully selected to avoid the melting of the inner structure of the composites during the survey.

#### 2.3.6. SSPCMs Density

The apparent bulk density of the composites was determined by weighing and sizing three cylindrical pellets of each sample. The true density was determined by helium pycnometry (Micromeritics Accupyc 1340).

#### 2.3.7. Thermal Degradation of SSPCMs Composites

Thermogravimetric analyses (TGAs) of SSPCMs and their raw materials were performed by using a TG 209 F1 Libra^®^ Thermogravimetric Analyzer from NETZSCH. The used conditions for the analyses were a heating rate of 10 °C/min from room temperature to 700 °C under nitrogen atmosphere.

#### 2.3.8. Thermal Properties

Differential scanning calorimetry (DSC) was used to determine the transition/melting temperatures, as well as the corresponding latent heats of NPG, Docosane and the obtained SSPCMs. A power-compensation DSC Q2500 from TA Instruments was employed with closed aluminum crucibles. The mass of the samples was ca. 9 mg. Argon (50 mL/min) was employed as purge gas. Each sample was submitted to three cycles of heating and cooling from 10 °C to 80 °C, with a heating/cooling rate of 5 °C/min. Additionally, the transient Hot Disk method was used to determine the thermal properties of the studied composites at room temperature, i.e., when the Docosane is in solid phase [25]. The measurements were carried out on cylindrical-shaped samples (diameter: 22 mm, thickness: 5 mm). A TPS 2500 S instrument equipped with a Kapton sensor of 2.001 mm in diameter (sensor type 7577) was employed for measurements. The power applied to the rear face of the sample was 20 mW during 20 s. One can notice that additional tests were performed with different heating power (from 20 to 40 mW) to ensure the reproducibility of the experiments. The recorded data were processed using the Hot Disk Software (version 7.4.0.10) and confirmed using the model proposed by Jannot and Acem [26].

## 3. Results and Discussion

### 3.1. NPG Crystal Sizes

**Figure 1:**
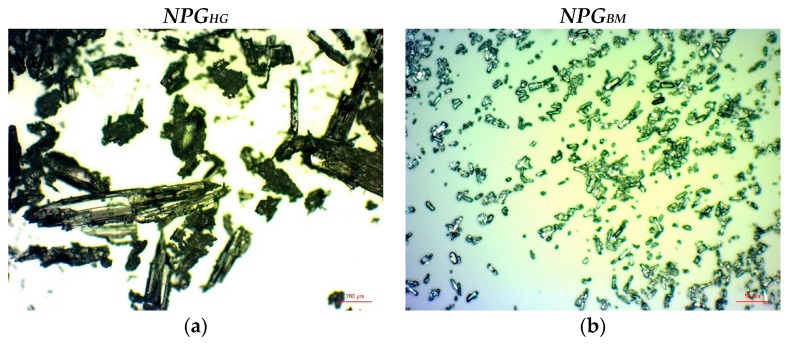
Optical microscope images of NPG crystals of (**a**) *NPH_HG_* and (**b**) *NPG_BM_* obtained at x5 and x20 magnification, respectively.

Figure 1 shows the optical microscope pictures of NPG crystals obtained after two milling methods, mortar (*NPG_HG_*) and ball-milling (*NPG_BM_*). As expected, the use of ball-milling significantly decreases the NPG crystal size compared to the manual grinding process due to the higher milling energy delivered by the mechanical system. From these pictures the NPG crystal size distribution was calculated for each case by using the software ImageJ2.0.

**Figure 2:**
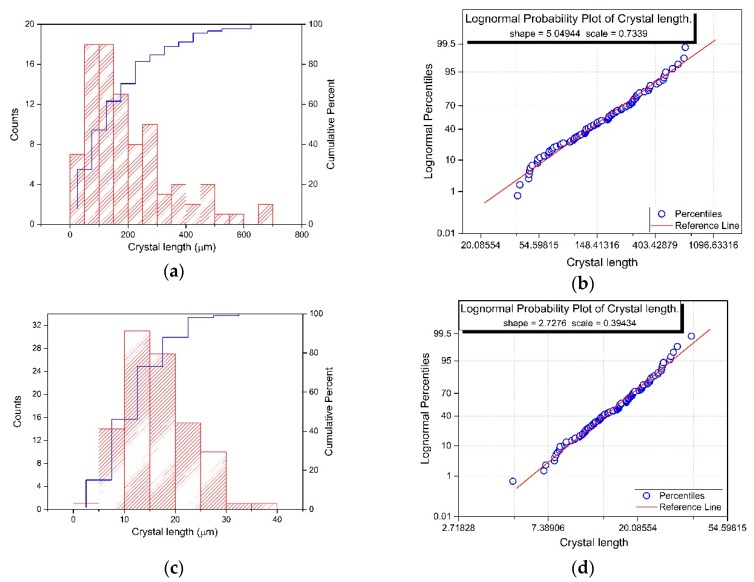
(**a**) *NPG_HG_*: Histogram and cumulative probabilities of NPG crystal size distribution; (**b**) *NPG_HG_*: lognormal probability plot of NPG crystal size; (**c**) *NPG_BM_*: Histogram and cumulative probabilities of NPG crystal size distribution; (**d**) *NPG_BM_*: Lognormal probability plot of NPG crystal size.

The crystal size distribution, depicted in Figure 2, presents a log-normal distribution in both cases, given by the density function *p*(*x*):

(1) $$p\left(x\right)=\frac{1}{\sigma x\sqrt{2\pi }}exp\left(-\frac{{\left(\ln\left(x\right)-\mu \right)}^{2}}{2\sigma^{2}}\right),$$

where *µ* is the shape scale parameter and *σ* the scale parameter (mean and standard deviation of the natural logarithm of the variable, respectively).

From these parameters the arithmetic mean and the arithmetic standard deviation can be calculated by Equation (2):

(2) $$Mean=\exp\left(\mu +\sigma^{2}/2\right),$$

and Equation (3):

(3) $$Standard\;Deviation=\sqrt{\left[\exp\left(\sigma^{2}\right)-1\right]\cdot \exp\left(2\mu +\sigma^{2}\right)}.$$

Therefore, *NPG_HG_* exhibits a mean NPG crystal size of 204.13 μm with a standard deviation of 172.44 µm whereas *NPG_BM_* has a mean NPG crystal size of 16.53 μm with a standard deviation of 6.78 µm, crystal size which is up to ten times lower than the displayed by *NPG_HG_*.

### 3.2. NPG-Docosane Wettability

**Figure 3:**
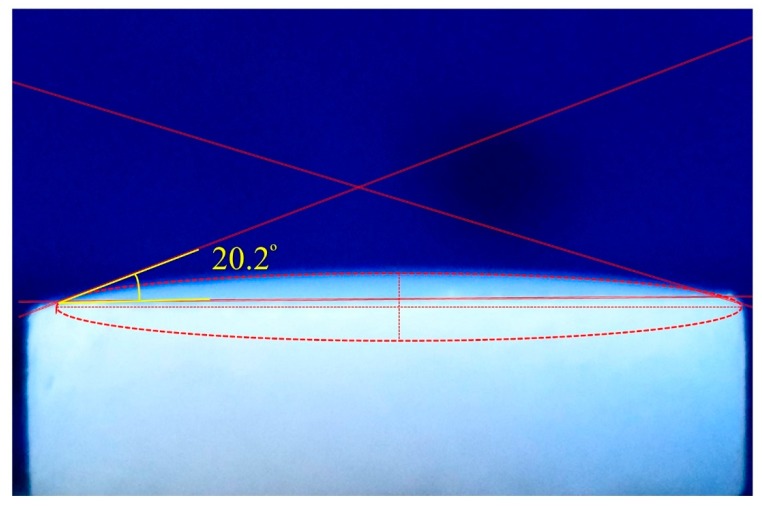
Contact angle of Docosane on the NPG surface at 70 °C.

A solid surface is deemed to be wetted if a liquid spreads over the surface without the formation of droplets. The contact angle of a liquid droplet on a solid substrate is a simple method for defining the wettability of the solid by the liquid. If this angle is less than 30° the solid-liquid system presents “good wetting” properties [27]. Figure 3 shows the contact angle of Docosane on the NPG surface at 70 °C (avoiding the solidification of Docosane).

As can be seen, the Docosane droplet presents a contact angle of 20°, which indicates a high wettability of NPG by Docosane at the measuring conditions.

The small contact angles on liquid-solid interfaces are due to the higher attraction of liquids by the solid surfaces, (work of adhesion), than to other liquid molecules (work of cohesion). The balance of these two opposing forces, work of adhesion and work of cohesion, in a porous media results in a curved liquid-gas interface which drives to a pressure difference across the interface, which in turn causes the phenomenon of capillarity [28,29,30]. This capillary force is the responsible of the retention of liquids in porous media.

### 3.3. Leakage Test

**Figure 4:**
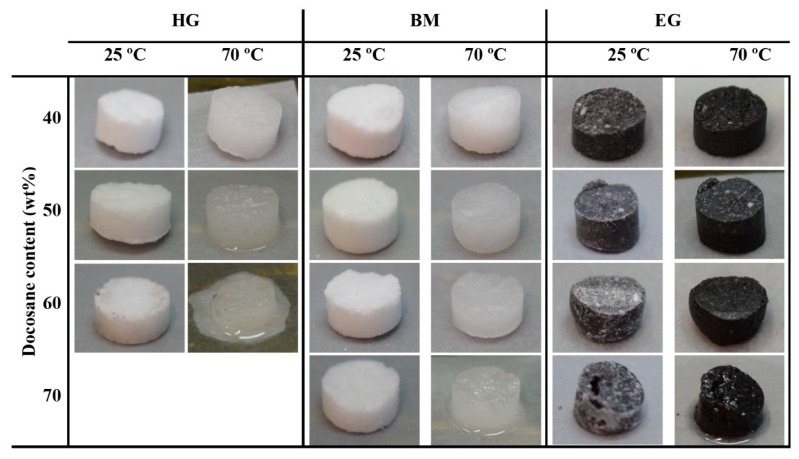
Obtained NPG-Docosane samples at 25 °C (left) and after 1 h at 70 °C (right).

In order to study the stabilization effect of the NPG crystal size on the final composite, samples having different Docosane contents were heated from 25 °C up to 70 °C, keeping the final temperature constant for 1 h. Moreover, the incorporation of expanded graphite as additional stabilizer was explored. Figure 4 shows the picture of the composites before (at 25 °C) and after the leakage test (at 70 °C).

**Figure 5:**
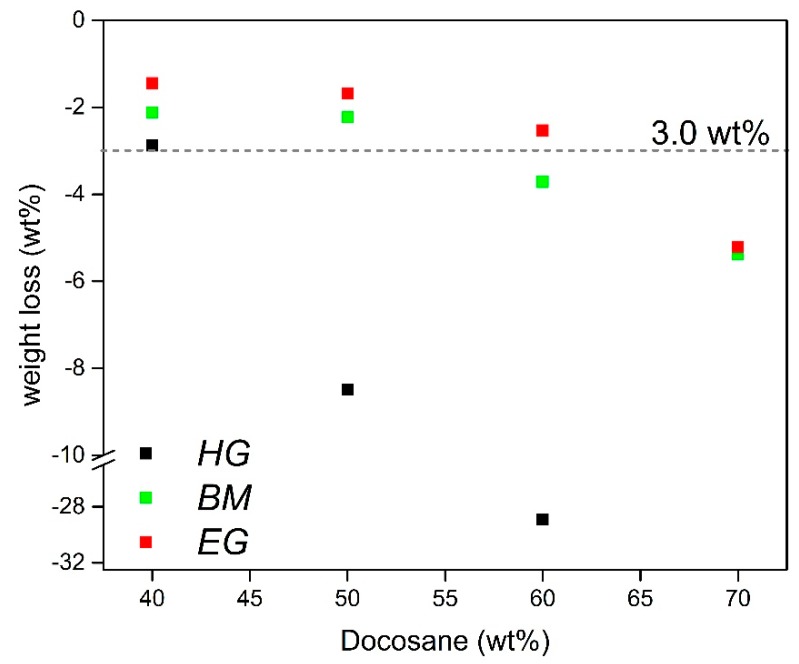
Weight loss of composites at 70 °C attributed to the leakage of Docosane.

It is plain to see that the increase of Docosane content decreases the stability of the final composite, leading to leakage of PCM at higher temperatures than its melting point. Moreover, it is clear from the pictures that lower NPG crystal sizes (*NPG_BM_*) allow the incorporation of larger amount of Docosane while keeping the shape of the final composite (*BM* composites). In the same way, samples containing expanded graphite also exhibit better stability than that shown by both *HG* and *BM* composites. The weight loss of the samples after leakage test is depicted in Figure 5.

The results gathered in the graph corroborate the behaviours observed in the pictures, whereas the PCM leakage of *HG* samples sharply increases from 50 wt% of Docosane content, the loss of PCM in *BM* and *EG* samples remains below 6 wt% for Docosane contents up to 70 wt%.

This behaviour can be related to the phenomenon of capillarity. Indeed, the most important adhesion forces in colloidal systems are van der Waals forces, electrostatic forces, and capillary forces. However, the later ones dominate over van der Waals and electrostatic forces by several order of magnitude in the micro-scale range [31,32,33]. Therefore, the ability of NPG micro-particles to hold the Docosane in its interstices mainly relies on the capillary forces, which grow with the pressure difference across the liquid-gas interface (∆*P*). For a hemispherical liquid-gas interface having radius of curvature *R*, the pressure difference is given by the Young-Laplace equation:

∆*P* = 2·*σ*/*R*, (4)

where *σ* is the interfacial surface tension [28]. Therefore, smaller NPG crystal sizes (*BM* samples) would lead to smaller porous sizes, promoting smaller radius of curvature and hence stronger capillary forces able to retain higher Docosane content. On the other hand, the incorporation of expanded graphite slightly enhances the stability of the composites due to its high sorption capacity to paraffin, as previously reported by Zhang P. et al. [34].

Taking a Docosane loss of 3 wt% as threshold to consider a composite as stable, the maximum amount of solid-liquid PCM reached in each case keeping the stability was 40, 50 and 60 wt% for samples *HG* (*HG_40_*), *BM* (*BM_50_*) and *EG* (*EG_60_*), respectively.

### 3.4. Inner Morphology

**Figure 6:**
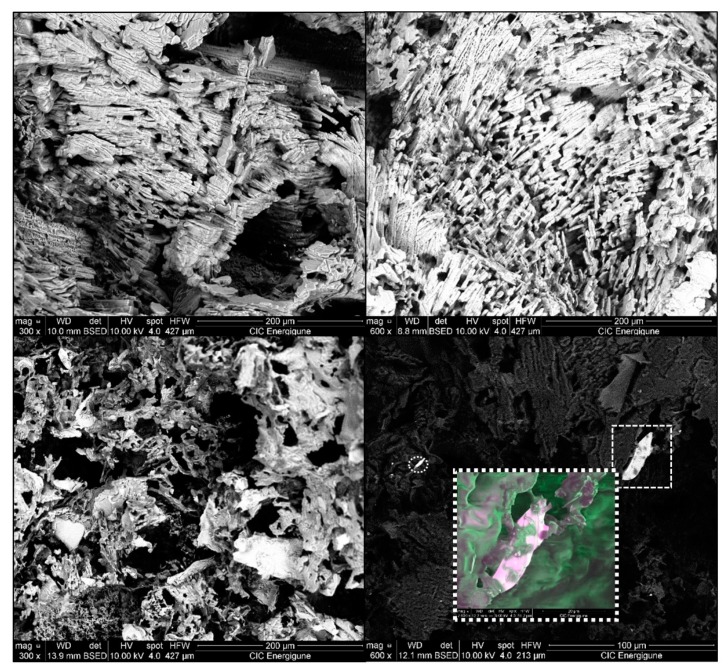
SEM images of *HG_40_* (top left corner), *BM_40_* (top right corner) and *EG_40_* (bottom pictures).

SEM photographs of the structure of composites *HG_40_*, *BM_40_* and *EG_40_* are shown in Figure 6.

As can be seen, both *HG_40_* and *BM_40_* samples present a complex cross-lamellar microstructure related to the presence of NPG crystals around which Docosane is perfectly located adopting the crystal shape. Therefore, due to its longer NPG crystals, foils of *HG_40_* are greater than those shown by *BM_40_* which is composed by NPG crystals sizes up to ten times lower than *HG_40_*. The shorter foils exhibited by *BM_40_* leads to smaller interstice sizes, which is in accordance with the above commented better retention of the Docosane by *BM* samples due to stronger capillary forces than the ones exhibited by *HG* composites.

On the contrary, the presence of expanded graphite in *EG_40_* modifies the morphology of the microstructure, changing from crystal shape where the edges of each foil are well defined to an amorphous shape without lamellas. This microstructure change can be explained observing the role of expanded graphite in the composite in Figure 6 bottom right corner, where, in the general picture, the expanded graphite particles are observed as bright flakes, corresponding to the contour of the typical worm-like shape of that particles [35]. A magnification of one of the expanded graphite particle is exposed in the coloured box, where the pictures taken with LFD and BSED detectors have been combined (50% of each one), noticing that these particles are also acting as binders of the Docosane, leading to an irregular shape and increasing the stability of the final composite, as has been observed in the leakage test.

### 3.5. Composite Density

The true density and the bulk density of the composites are shown in Table 2. As expected, the true density decreases with the Docosane content due to its lower density (0.778 g/cm^3^ at 25 °C) compared to the exhibited by the NPG (1.060 g/cm^3^ at 25 °C). The impact of the expanded graphite on the composites is reflected in their bulk densities. For equal Docosane content, the presence of expanded graphite increases the bulk density due to its previously commented binder effect which reduces the air voids in the sample, therefore the density evolves as follows: *EG_50_* > *BM_50_* and *EG_40_* > *HG_40_* > *BM_40_*.

As previously commented, the combination of polyhydric alcohols and paraffin not only could boost the thermal energy storage of the first due to the high latent heat of the paraffin, but also provides shape stabilization at the time that increases the density of the material due to the higher density of polyhydric alcohols compared to the one exhibited by alkanes [36]. This higher density also leads to greater volumetric energy storage densities, overall considering that the component which provides the higher density also presents a high phase transition latent heat.

### 3.6. Crystallization Properties of NPG/Docosane Composites

**Figure 7:**
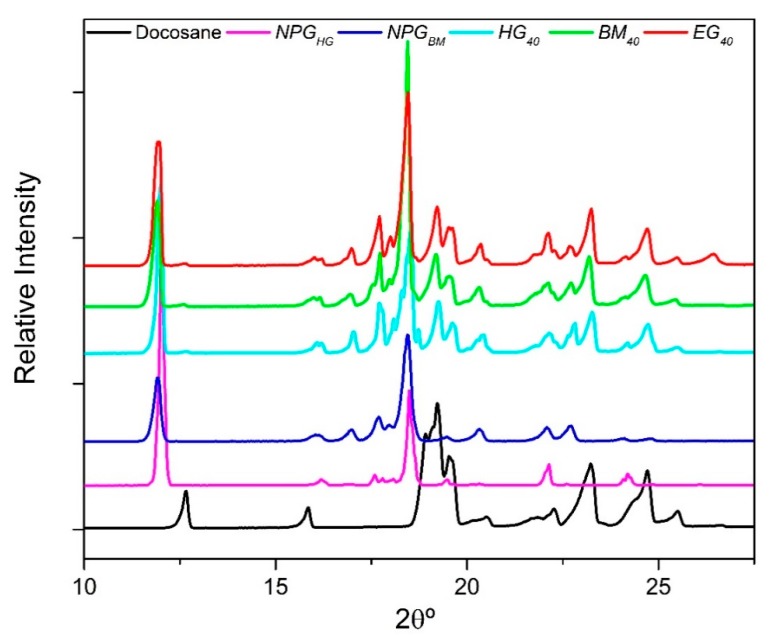
XRD diffractogram of Docosane, NPG and their mixtures containing 40 wt% of Docosane.

Figure 7 shows the XRD patterns of the raw materials, Docosane and NPG (*NPG_HG_* and *NPG_BM_*), as well as the diffractograms related to the composites containing 40 wt% of Docosane (*HG_40_*, *BM_40_* and *EG_40_*).

As shown in Figure 7, Docosane and NPG present different diffraction peaks. At the analysis conditions (25 °C) Docosane has a series of main diffraction peaks at 12.6°, 15.9°, 18.6°–19.8°, 23.3° and 24.7°, whose pattern corresponds to the characteristic triclinic phase of Docosane at low temperature [37]. On the other hand, the main peaks of *NPG_HG_* pattern appear at 11.96°, 16.12°, 17.7°, 18.45°, 19.5°, 22.14° and 24.2° [22,38], corresponding to its reported monoclinic structure with space group of P2_1/n_ at low temperature, nevertheless, the peaks of *NPG_BM_* pattern slightly broaden out and shift to lower 2θ° values than the showed by the *NPG_HG_* pattern. This behaviour is related to a decrease in the crystallite size and the appearance of microstrain and dislocations in the powder structure derived from the mechanical milling process [39].

The main characteristic peaks of each raw compounds also appear in the diffractogram of both composites *HG_40_*, *BM_40_* and *EG_40_*, moreover, as in the previous case, the mechanical milling process used in *BM_40_* and *EG_40_* shifts their peaks associated to the NPG pattern. Finally, the absence of new peaks in all the composites suggests that no chemical interaction occurs between NPG and Docosane and that their main crystal structures were not affected after they were mixed.

### 3.7. Thermal Stability

**Figure 8:**
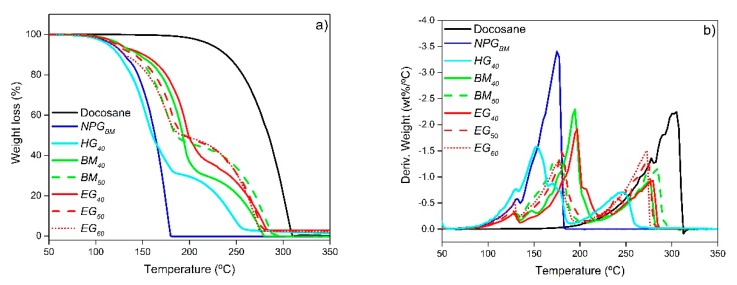
(**a**) TGA and (**b**) DTGA profiles of the samples and their raw materials.

Figure 8 presents the thermogravimetric analysis (weight loss and derivative weight loss) of the obtained composites and their raw materials.

Regarding the pure components, whereas the weight loss of Dosocane occurs between 175 to 310 °C, NPG has two degradation peaks, the first one below 135 °C related to the adsorbed water due to its hygroscopicity, and the main peak between 135 to 180 °C attributed to its thermal degradation. It should be pointed out that, both *NPG_BM_* and *NPG_HG_* exhibit similar degradation profiles, therefore only *NPG_BM_* profile is plotted. In line with pure components, the composites show three weight loss peaks (Figure 8b). The first weight loss occurs below 135 °C for all the samples and, as has been previously commented, corresponds to the adsorbed water by the NPG. The second degradation step, related to the NPG, begins at 135 °C, but continues until different temperatures as function of the Docosane content and the type of shape-stabilizer material. This way, *HG_40_*, with the highest NPG crystal size (*NPG_HG_*) and worst NPG-Docosane interaction has the earliest thermal degradation, finishing at 190 °C. On the contrary, the increase of the NPG-Docosane contact in the samples composed by *NPG_BM_* improves the stability of the composites delaying their thermal degradation until temperatures between 195 to 222 °C. Likewise, for the same Docosane content, the presence of expanded graphite retards the end of the NPG thermal degradation due to its binder effect, 222 °C for *EG_40_* against 217 °C for *BM_40_* or 209 °C for *EG_50_* against 204 °C for *BM_50_*. The third and last weight loss, attributed to Docosane, goes forward in relation to the decomposition temperature of the pure compound. This displacement could be related to the vaporization of the NPG, which would drive the degradation of Docosane. The remaining residue observed in *EG_40_* (2.84 wt%), *EG_50_* (2.63 wt%) and *EG_60_* (2.24 wt%) samples corresponds to the added expanded graphite.

### 3.8. Thermal Properties

#### 3.8.1. DSC Analysis

**Figure 9:**
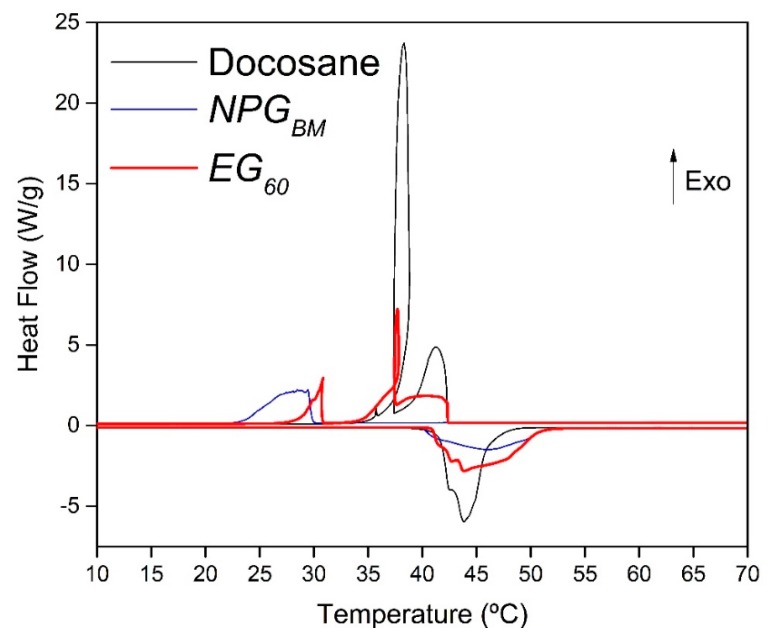
DSC thermogram (heat flux vs. temperature) obtained for NPG, Docosane and EG60. Negative heat flux values correspond to endothermic phenomena whereas positive values represent exothermic phenomena.

Phase transition temperatures and latent heats of the composites and their raw materials were studied by DSC analyses. Figure 9 shows the thermograms of *NPG_BM_*, *Docosane* and *EG_60_* as a representative example of the samples, whereas the obtained results for all the composites are depicted in Table 3. Both the thermograms showed in Figure 9 and the values gathered in Table 3 correspond to the third cycle of the DSC because no significant differences were found regarding the two previous heating/cooling cycles.

It can be seen from Figure 9 that the transition of *NPG* from monoclinic crystal structure to FCC crystal [40] starts at 42 °C. However, during the cooling, the transition from FCC to monoclinic structure is delayed 12 °C, appearing at 30 °C. In the case of *Docosane*, the DSC thermogram shows two overlapped peaks, both on melting and on crystallization. During the heating, the first one (41–42 °C) is related to a solid-solid transition from a crystalline to a disordered phase [41], then, melting takes place at 43–44 °C. On the contrary, in the cooling step the first peak corresponds to the crystallization of Docosane (42–37 °C), followed by the solid-solid transition at 37 °C.

The signature of both NPG and Docosane is appreciated in the heat flow profile of *EG_60_*. In the endothermic period, three overlapped peaks appear at the same temperature range than those observed for NPG and Docosane. In the same way, during the exothermic step, the phase transitions of raw materials come into view in *EG_60_*. The two first peaks correspond to the crystallization and the solid-solid transition of Docosane, whereas the third one (at the lowest temperature) is linked to the transition of NPG from FCC to the monoclinic crystal structure. The crystallization behavior of Docosane within the composite does not differ from that of single Docosane, whereas the “monoclinic to FCC” transition of the NPG within the composite shifts toward higher temperature than the exhibited by the pure NPG.

The phase transition temperatures and the enthalpy changes calculated from the DSC thermograms are reported in Table 3. Enthalpy values in Table 3 only account for latent heat because an integral tangential interpolated baseline was employed for the integration of the phase transition peaks of the thermograms. It must be noticed that for NPG-Docosane composites: (i) the two onset temperatures recorded in cooling are related to the initial crystallization temperature of Docosane (1st T_onset_) and the initial transition temperature of NPG (2nd T_onset_); (ii) the latent heat values reported in cooling include both the latent heat of Docosane and the latent heat of “monoclinic to FCC” transition of NPG, and (iii) the theoretical values of latent heat (Δ*H_th_*) have been calculated by using the mixing rule showed in Equation (5), where *X_NPG_* and *X_Docosane_* are respectively the mass fraction of NPG and Docosane in the final composite, taking into account the presence of EG where applicable, and ∆*H_NPG_* and ∆*H_Docosane_* are the phase transition latent heat of pure *NPG* and *Docosane*, respectively, obtained in heating or cooling as required.

(5) $$\Delta H_{th}=\Delta H_{NPG}\cdot X_{NPG}+\Delta H_{Docosane}\cdot X_{Docosane}$$

One can notice that the obtained temperature and latent heat values for raw materials (NPG and Docosane) are in agreement with the literature [42]. As expected, the higher the Docosane content, the higher the latent heat of the composites since the latent heat of pure Docosane is twice as high as the transition heat of pure NPG. It is also pointed out that the same main observations reported for *EG_60_* can be applied to the other composites, whose heat flow profiles exhibited the characteristic phase transition temperatures presented in their raw materials. Moreover, there is a good agreement between the latent heat values of the composites and the values calculated by the mixing rule. These results suggest that the phase change properties of NPG and Docosane are not modified in the composites, which together with the XRD analysis allow to conclude that there is good chemical compatibility between NPG and Docosane and that they are non-miscible with each other.

The election of the proper n-alkane and polyhydric alcohol with the same phase transition temperature allows the combination of their latent/transition heats, working as a single compound with high latent heat and stable shape. In this way, the maximum latent heat achieved in this paper is 210.74 J/g for *EG_60_* (heating stage), value which is much higher than those latent heats previously reported in literature for conventional shape-stabilized PCMs in the same working temperature range. Table 4 provides a summary of several reported SSPCMs and their phase change, latent heats having a similar range of working temperatures than the exposed by *EG_60_*.

It is plain to see that, even for higher solid-liquid PCM content, the use of a stabilizer matrix having thermal energy storage properties (NPG) boosts the final latent heat of the composite in comparison with conventional SSPCMs.

#### 3.8.2. Hot Disk

The transient Hot Disk method was used to determine the thermal conductivities (*k*) and thermal diffusivities (*α*) of the studied composites at room temperature (25 °C). The estimated parameters are reported in Table 5.

As can be seen, in the samples without *EG* the increase of the Docosane content enhances the thermal conductivity of the SSPCM (*BM_50_*) due to the higher *k* of pure Docosane (0.28 W/m·K) compared to NPG (0.25 W/m·K). On the other hand, both thermal conductivity and thermal diffusivity sharply increase when *EG* is incorporated. The effect of the expanded graphite on the heat transfer properties of the SSPCMs is related to three phenomena, the higher thermal conductivity of the expanded graphite itself (1–7 W/m·K) [55], the creation of a continuous network between the particles of expanded graphite which facilitates the heat transfer [3,34] and also the above commented increase of the bulk density of the materials that have less air voids acting as insulating pores [56]. Therefore, due to these three contributions and to the fact that the spatial distribution of *EG* across the samples (continuous network) has not been controlled, it is adventurous to find a correlation within the *k* values of the three samples (*EG_40_*, *EG_50_*, and *EG_60_*), hence it can be merely asserted that the presence of *EG* sharply increases *k*. As the thermal conductivity, the thermal diffusivity rises with the *EG* addition. In Hot Disk method, thermal sensitivity to α is much lower than to k [26], therefore, the growth in terms of α from *EG_40_* to *EG_60_* should not be taken as reliable base for any additional assumption.

Consequently, the addition of *EG* in our samples not only allows to increase their stability and maximum Docosane retention, but also allows to improve their heat transfer capacity.

## 4. Conclusions

Their chemical compatibility, good wettability and similar range of phase transition temperatures allow the combination of NPG and Docosane for working as a SSPCM having high thermal energy storage capacity. A maximum Docosane content of 60 wt% was achieved through the NPG crystal size control and the addition of 2.5 wt% of *EG*. The latent heat obtained in these conditions (210.74 J/g) was much higher than those latent heats exhibited by conventional SSPCMs in the same working temperature range. Moreover, the addition of *EG* sharply increased both the thermal conductivity and the thermal diffusivity of the composites.

These results demonstrate the feasibility of using polyalcohols with energetic solid-solid phase transitions as an active supporting media in SSPCMs where the solid-liquid PCM is an n-alkane. Furthermore, the use of the solid-solid PCM itself as an external coating of the SSPCM would allow to increase the solid-liquid PCM content beyond the current maximum value of 60 wt%. The range of working temperatures of alkanes (40 °C to 115 °C) with high latent heats (200 J/g to 300 J/g) and the possibility to tailor the transition temperature of polyalcohols by means of binary and ternary mixtures open a wide variety of interesting opportunities to combine them, increasing the energy density of polyalcohols while lowering the cost and preserving the advantages of SSPCMs.

## Figures and Tables

**Table 1:** Main properties of Neopentyl Glycol (NPG) and Docosane.

	CAS Number	*T_t_* (°C)	Δ*H* (J/g)	*ρ*_25°C_ (g/cm^3^)	*k*_30°C_ (W/m·K)
**Neopentyl Glycol**	126-30-7	48 ^1^	126.33 ^3^	1.060	0.25 [20]
**Docosane**	629-97-0	42–45 ^2^	259.14 ^3^	0.778	0.28 [21]

^1^ Transition temperature of solid-solid phase change [20]. ^2^ Transition temperature of solid-liquid phase change given by the supplier. ^3^ Measured in this work as described below.

**Table 2:** True density and apparent bulk density of the stable composites.

Sample	True Density (g/cm^3^)	Bulk Density (g/cm^3^)
***HG_40_***	1.014	0.878
***BM_40_***	0.993	0.843
***BM_50_***	0.990	0.810
***EG_40_***	1.013	0.894
***EG_50_***	0.999	0.856
***EG_60_***	0.972	0.799

**Table 3:** True density and apparent bulk density of the stable composites.

Sample	Heating				Cooling			
	T_onset_ (°C)	T_offset_ (°C)	Δ*H* (J/g)	Δ*H_th_* (J/g)	1st T_onse_t (°C)	2nd T_onset_ (°C)	Δ*H* (J/g)	Δ*H_th_* (J/g)
***Docosane***	41.64	46.36	259.14		42.29		258.83	
***NPG***	40.08	52.31	126.33		30.01		113.96	
***HG_40_***	40.50	51.24	177.04	179.45	42.14	30.38	172.94	171.91
***BM_40_***	40.70	53.21	176.67	179.45	42.03	30.35	170.51	171.91
***BM_50_***	40.56	48.42	190.65	192.74	42.20	30.83	187.67	186.40
***EG_40_***	40.75	50.41	165.40	176.30	42.30	30.66	160.70	169.06
***EG_50_***	40.70	50.02	196.12	189.58	42.40	30.21	193.30	183.55
***EG_60_***	40.93	51.18	210.74	202.86	42.38	30.82	206.93	198.03

**Table 4:** Summary of some reported SSPCMs with a phase transition around 40 °C.

Type of Composite	Composition	PCM (wt%)	*T_t_* (°C)	Δ*H* (J/g)
SSPCM	High-density polyethylene + Pentacosane [43]	74	37.5–54	121
SSPCM	High-density polyethylene + Refined Paraffin [44]	75	40–55	157
SSPCM	Styrene-butadiene-styrene + Paraffin [45]	80	56–58	165.2
Solid-solid Polyurethane + SSPCM	PolyurethanePCM + n-octadecane/n-eicosane [16]	25	20–65	141.2
SSPCM	HDPE + P2 Paraffin + 3wt% Expanded graphite [46]	77	45.2–55.7	162.2
Eutectic Solid-solid PCM	Pentaglycerine + Neopentylglycol [47]	70 ^1^	44–74	117.9 ^2^
SSPCM	Poly(vinyl chloride) + Lauric acid [48]	50	38.8	91.6
SSPCM	Poly(vinyl chloride) + Malonic acid [48]	50	49.2	99.2
SSPCM	Poly(vinyl alcohol) + Lauric acid [49]	50	39.8	96.4
SSPCM	Poly(vinyl alcohol) + Malonic acid [49]	50	50.2	105.3
Solid-solid PCM	Cellulose graft poly(ethylene glycol) copolymers [50]	77.4	42.8	92
SSPCM	Styrene maleic anhydride copolymer + Malonic acid [51]	85	51.75	176.49
SSPCM	Styrene maleic anhydride copolymer + Lauric acid [51]	85	41.48	160.83
SSPCM	Poly (methyl methacrylate) + Malonic acid [52]	80	51	166.56
SSPCM	Poly (methyl methacrylate) + Lauric acid [52]	80	40.96	149.55
SSPCM	Acrylic cationic resin + Malonic acid [53]	70	51.44	135.62
Encapsulation + SSPCM	Styrene-ethylene-butylene-styrene + Paraffin IGI 422 [54]	75	44–55	189

^1^ PCM content refers to the NPG content. ^2^ Calculated from their theoretical latent heats.

**Table 5:** Thermal conductivities (*k*) and thermal diffusivities (*α*) of the studied composites.

Sample	*k* (W/m·K)	*α* (mm^2^/s)
***HG_40_***	0.277	0.221
***BM_40_***	0.276	0.174
***BM_50_***	0.295	0.197
***EG_40_***	0.433	0.332
***EG_50_***	0.519	0.345
***EG_60_***	0.450	0.467

## References

[1] Sarbu I., Sebarchievici C. (2018). A comprehensive review of thermal energy storage. Sustainability.

[2] Sutterlin W.R. (2014). Phase Change Materials, A Brief Comparison of Ice Packs, Salts, Paraffins, and Vegetable-Derived Phase Change Materials.

[3] Nazir H., Batool M., Bolivar Osorio F.J., Isaza-Ruiz M., Xu X., Vignarooban K., Phelan P., Inamuddin, Kannan A.M. (2019). Recent developments in phase change materials for energy storage applications: A review. Int. J. Heat Mass Transf..

[4] Jamekhorshid A., Sadrameli S.M., Farid M. (2014). A review of microencapsulation methods of phase change materials (PCMs) as a thermal energy storage (TES) medium. Renew. Sustain. Energy Rev..

[5] Huang X., Zhu C., Lin Y., Fang G. (2019). Thermal properties and applications of microencapsulated PCM for thermal energy storage: A review. Appl. Therm. Eng..

[6] Chen C., Liu W., Wang Z., Peng K., Pan W., Xie Q. (2015). Novel form stable phase change materials based on the composites of polyethylene glycol/polymeric solid-solid phase change material. Sol. Energy Mater. Sol. Cells.

[7] Guo Q., Wang T. (2014). Preparation and characterization of sodium sulfate/silica composite as a shape-stabilized phase change material by sol-gel method. Chin. J. Chem. Eng..

[8] Cheng W.L., Zhang R.M., Xie K., Liu N., Wang J. (2010). Heat conduction enhanced shape-stabilized paraffin/HDPE composite PCMs by graphite addition: Preparation and thermal properties. Sol. Energy Mater. Sol. Cells.

[9] Sari A., Alkan C., Karaipekli A., Uzun O. (2010). Poly(ethylene glycol)/poly(methyl methacrylate) blends as novel form-stable phase-change materials for thermal energy storage. J. Appl. Polym. Sci..

[10] Umair M.M., Zhang Y., Iqbal K., Zhang S., Tang B. (2019). Novel strategies and supporting materials applied to shape-stabilize organic phase change materials for thermal energy storage—A review. Appl. Energy.

[11] Li M., Shi J. (2019). Review on micropore grade inorganic porous medium based form stable composite phase change materials: Preparation, performance improvement and effects on the properties of cement mortar. Constr. Build. Mater..

[12] Tang B., Cui J., Wang Y., Jia C., Zhang S. (2013). Facile synthesis and performances of PEG/SiO_2_ composite form-stable phase change materials. Sol. Energy.

[13] Serrano A., Martín del Campo J., Peco N., Rodriguez J.F., Carmona M. (2019). Influence of gelation step for preparing PEG–SiO_2_ shape-stabilized phase change materials by sol-gel method. J. Sol-Gel Sci. Technol..

[14] Gao H., Wang J., Chen X., Wang G., Huang X., Li A., Dong W. (2018). Nanoconfinement effects on thermal properties of nanoporous shape-stabilized composite PCMs: A review. Nano Energy.

[15] Fallahi A., Guldentops G., Tao M., Granados-Focil S., Van Dessel S. (2017). Review on solid-solid phase change materials for thermal energy storage: Molecular structure and thermal properties. Appl. Therm. Eng..

[16] Chen K., Yu X., Tian C., Wang J. (2014). Preparation and characterization of form-stable paraffin/polyurethane composites as phase change materials for thermal energy storage. Energy Convers. Manag..

[17] Mishra A., Talekar A., Chandra D., Chien W.M. (2012). Ternary phase diagram calculations of pentaerythritol-pentaglycerine-neopentylglycol system. Thermochim. Acta.

[18] Gunasekara S.N., Pan R., Chiu J.N., Martin V. (2016). Polyols as phase change materials for surplus thermal energy storage. Appl. Energy.

[19] Zgardzińska B., Gorgol M. (2014). Influence of pressure on the size of free volumes in some waxes. Acta Phys. Pol. A.

[20] Wang J., Chen G., Jiang H. (1999). Theoretical study on a novel phase change process. Int. J. Energy Res..

[21] Vélez C., Khayet M., Ortiz De Zárate J.M. (2015). Temperature-dependent thermal properties of solid/liquid phase change even-numbered n-alkanes: N-Hexadecane, n-octadecane and n-eicosane. Appl. Energy.

[22] Chandra D., Day C.S., Barrett C.S. (1993). Low- and high-temperature structures of neopentylglycol plastic crystal. Powder Diffr..

[23] Rueden C.T., Schindelin J., Hiner M.C., DeZonia B.E., Walter A.E., Arena E.T., Eliceiri K.W. (2017). ImageJ2: ImageJ for the next generation of scientific image data. BMC Bioinform..

[24] Brugnara M. (2010). Contact Angle Plugin for ImageJ.

[25] Gustafsson S.E. (1991). Transient plane source techniques for thermal conductivity and thermal diffusivity measurements of solid materials. Rev. Sci. Instrum..

[26] Jannot Y., Acem Z. (2007). A quadrupolar complete model of the hot disc. Meas. Sci. Technol..

[27] Arkles B. (2015). Hydrophobicity, Hydrophilicity and Silane Surface Modification.

[28] Or D., Tuller M. (2005). Capillarity.

[29] Schrader M.E. (1995). Young-Dupre Revisited. Langmuir.

[30] Packham D.E. (1996). Work of adhesion: Contact angles and contact mechanics. Int. J. Adhes. Adhes..

[31] Megias-Alguacil D., Gauckler L.J. (2010). Capillary and van der Waals forces between uncharged colloidal particles linked by a liquid bridge. Colloid Polym. Sci..

[32] Leroch S., Wendland M. (2013). Influence of capillary bridge formation onto the silica nanoparticle interaction studied by grand canonical Monte Carlo simulations. Langmuir.

[33] Neugebauer R., Koriath H.J., Van der Merwe A.F., Müller M., Matope S. Study on Applicability of Adhesive Forces for Micro-Material Handling in production technology. Proceedings of the ISEM 2011 Proceedings.

[34] Zhang P., Hu Y., Song L., Ni J., Xing W., Wang J. (2010). Effect of expanded graphite on properties of high-density polyethylene/paraffin composite with intumescent flame retardant as a shape-stabilized phase change material. Sol. Energy Mater. Sol. Cells.

[35] Wang W., Yang X., Fang Y., Ding J., Yan J. (2009). Preparation and thermal properties of polyethylene glycol/expanded graphite blends for energy storage. Appl. Energy.

[36] Kenisarin M.M., Kenisarina K.M. (2012). Form-stable phase change materials for thermal energy storage. Renew. Sustain. Energy Rev..

[37] Fu D., Su Y., Xie B., Zhu H., Liu G., Wang D. (2011). Phase change materials of n-alkane-containing microcapsules: Observation of coexistence of ordered and rotator phases. Phys. Chem. Chem. Phys..

[38] Zannetti R. (1961). The unit cell and space group of 2,2-dimethyl-1,3-propanediol. Acta Crystallogr..

[39] Mahmoud A.E., Wasly H.S., Doheim M.A. (2014). Studies of crystallite size and lattice strain in Al-Al_2_O_3_ powders by high-energy mechanical milling. J. Eng. Sci..

[40] Benson D.K., Burrows R.W., Webb J.D. (1986). Solid state phase transitions in pentaerythritol and related polyhydric alcohols. Sol. Energy Mater..

[41] Domanska U., Wyrzykowska-Stankiewicz D. (1991). Enthalpies of fusion and solid-solid transition of even-numbered paraffins C22H46, C24H50, C26H54 and C28H58. Thermochim. Acta.

[42] Pielichowska K., Pielichowski K. (2014). Phase change materials for thermal energy storage. Prog. Mater. Sci..

[43] Inaba H., Tu P. (1997). Evaluation of thermophysical characteristics on shape-stabilized paraffin as a solid-liquid phase change material. Heat Mass Transf..

[44] Hong Y., Xin-shi G. (2000). Preparation of polyethylene-paraffin compound as a form-stable solid-liquid phase change material. Sol. Energy Mater. Sol. Cells.

[45] Xiao M., Feng B., Gong K. (2002). Preparation and performance of shape stabilized phase change thermal storage materials with high thermal conductivity. Energy Convers. Manag..

[46] Sari A. (2004). Form-stable paraffin/high density polyethylene composites as solid-liquid phase change material for thermal energy storage: Preparation and thermal properties. Energy Convers. Manag..

[47] Font J., Muntasell J., Navarro J., Tamarit J.L., Lloveras J. (1987). Calorimetric study of the mixtures PE/NPG and PG/NPG. Sol. Energy Mater..

[48] Sari A., Kaygusuz K. (2006). Studies on poly(vinyl chloride)/fatty acid blends as shape-stabilized phase change material for latent heat thermal energy storage. Indian J. Eng. Mater. Sci..

[49] Sari A., Kaygusuz K. (2007). Poly(vinyl alcohol)/fatty acid blends for thermal energy storage. Energy Sources Part A Recovery Util. Environ. Eff..

[50] Li Y., Wu M., Liu R., Huang Y. (2009). Cellulose-based solid-solid phase change materials synthesized in ionic liquid. Sol. Energy Mater. Sol. Cells.

[51] Sari A., Alkan C., Karaipekli A., Önal A. (2008). Preparation, characterization and thermal properties of styrene maleic anhydride copolymer (SMA)/fatty acid composites as form stable phase change materials. Energy Convers. Manag..

[52] Alkan C., Sari A. (2008). Fatty acid/poly(methyl methacrylate) (PMMA) blends as form-stable phase change materials for latent heat thermal energy storage. Sol. Energy.

[53] Kaygusuz K., Alkan C., Sari A., Uzun O. (2008). Encapsulated fatty acids in an acrylic resin as shape-stabilized phase change materials for latent heat thermal energy storage. Energy Sources Part A Recovery Util. Environ. Eff..

[54] Peng S., Fuchs A., Wirtz R.A. (2004). Polymeric phase change composites for thermal energy storage. J. Appl. Polym. Sci..

[55] Afanasov I.M., Savchenko D.V., Ionov S.G., Rusakov D.A., Seleznev A.N., Avdeev V.V. (2009). Thermal conductivity and mechanical properties of expanded graphite. Inorg. Mater..

[56] Wang X., Guo Q., Zhong Y., Wei X., Liu L. (2013). Heat transfer enhancement of neopentyl glycol using compressed expanded natural graphite for thermal energy storage. Renew. Energy.

